# Supplementary figures and images for: Marcks and Marcks-like 1 proteins promote spinal cord development and regeneration in Xenopus
Source: eLife. 2024 Dec 12;13:e98277. doi: 10.7554/eLife.98277 (PMC11637466; doi:10.7554/eLife.98277)

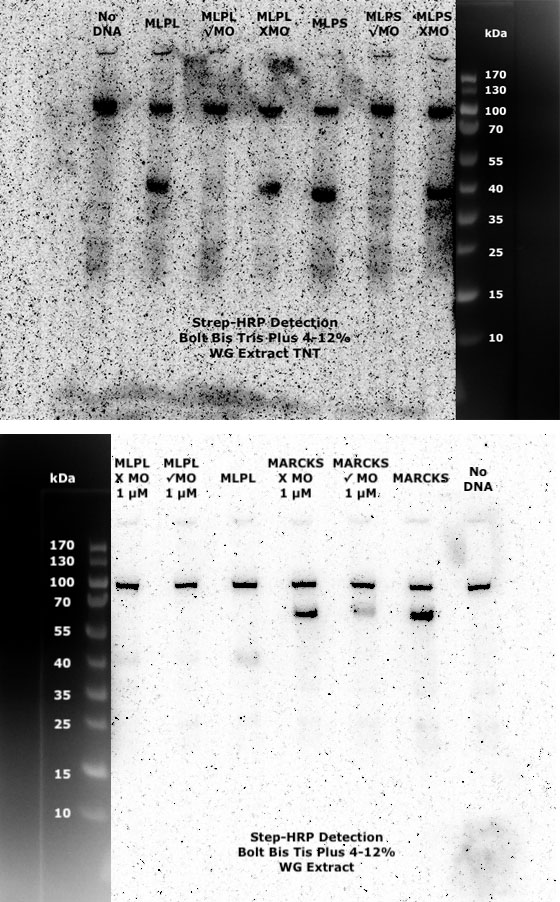

Supplement: Figure 2—figure supplement 1—source data 1. [file elife-98277-fig2-figsupp1-data1.zip › Figure 2ΓÇöfigure supplement 1ΓÇösource data 1.jpg]

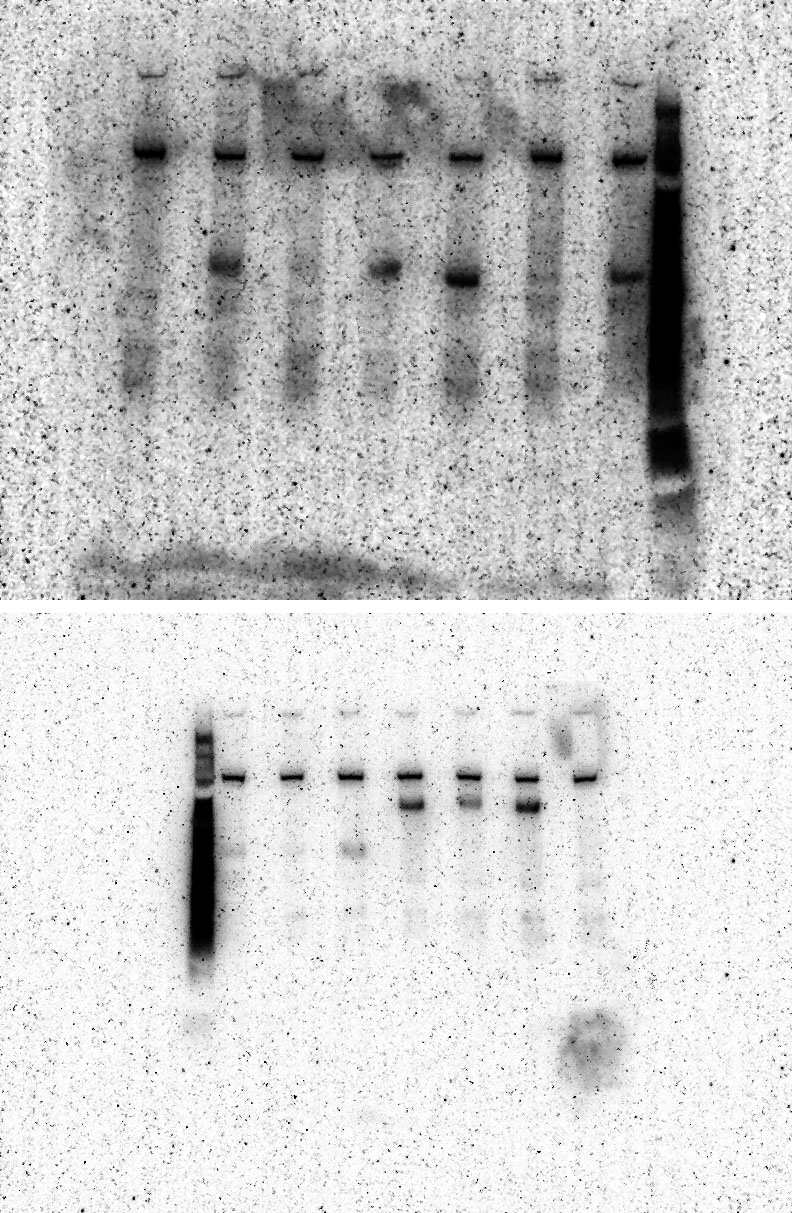

Supplement: Figure 2—figure supplement 1—source data 2. [file elife-98277-fig2-figsupp1-data2.zip › Figure 2ΓÇöfigure supplement 1ΓÇösource data 2.jpg]
